# Progressive Paraparesis and Spinal Glioblastoma in a Young Female

**DOI:** 10.7759/cureus.57593

**Published:** 2024-04-04

**Authors:** Andreea Anamaria Idu, Lucian Eva, Razvan-Adrian Covache-Busuioc, Luca-Andrei Glavan, Alexandru Vladimir Ciurea

**Affiliations:** 1 Department of Neurosurgery, Carol Davila University of Medicine and Pharmacy, Bucharest, ROU; 2 Department of Neurosurgery, Nicolae Oblu Clinical Emergency Hospital, Iasi, ROU

**Keywords:** neuro oncology, radiotherapy, multidisciplinary, chemotherapy, spinal glioblastoma

## Abstract

Glioblastoma, a primary brain tumor known for its adverse prognosis and aggressive nature, presents a significant challenge when occurring in the spinal cord. We report a case of a 20-year-old female with no prior medical history who developed progressive paraparesis and urinary retention, symptoms indicative of an intramedullary glioblastoma in the spinal cord. This case study delves into the clinical presentation, diagnostic process, and therapeutic interventions, highlighting the complexities encountered during the patient's treatment course. Despite the typically poor prognosis associated with glioblastoma, with an average survival rate of approximately 15 months post-diagnosis, our patient's initial response to adjuvant chemotherapy and radiotherapy extended her survival to 34 months. However, tumor recurrence ultimately led to a decision against aggressive treatment, reflecting the challenges in managing this devastating condition. This case emphasizes the importance of a multidisciplinary approach in the care of spinal glioblastoma patients, ranging from neurosurgery, anesthesiology and intensive care, radiology, oncology, anatomic pathology and nuclear medicine, underscoring the complexity of the disease, while highlighting the urgent need for ongoing research and innovation in neuro-oncology to improve treatment outcomes. The use of modern treatment techniques, including the potential role of nanomaterials for drug delivery, suggests avenues for future research. This case report contributes to the scarce literature on spinal glioblastoma, advocating for detailed documentation of cases to enhance understanding and treatment strategies for this formidable disease.

## Introduction

Glioblastoma represents the primary brain tumor characterized by the most adverse prognosis and is also the predominant type of brain neoplasm [1]. Its hallmark features include an infiltrative growth pattern, extensive vascularization, and a swift and aggressive clinical course [2]. According to the fifth edition of the World Health Organization (WHO) classification, glioblastoma is classified as grade 4, a designation that correlates with escalating degrees of aggressiveness and histopathological characteristics [3,4].

The annual incidence rate of glioma is estimated at approximately 5.26 instances per 100,000 individuals [5,6]. Among the malignant primary neoplasms of the central nervous system in adults, malignant astrocytomas represent the most prevalent category [7]. Furthermore, glioblastomas comprise about 60%-70% of all cases of malignant glioma [5,7,8].

However, primary spinal cord glioblastomas are extremely rare. For example, the incidence is for 9% of gliomas and 2.5% of all neuroglial intramedullary neoplasms [9]. In a population-based analysis conducted in Norway, primary spinal glioblastomas were identified as exceedingly uncommon neoplasms, comprising merely 0.2% of glioblastoma instances and 1.5% of all primary spinal cord tumors [10].

Individuals in the first two decades of life exhibit a heightened susceptibility to the development of this particular tumor phenotype, with the cervical spine identified as the predominantly affected anatomical region. Specifically, the lower segments of the cervical spine are most frequently implicated, followed by the thoracic spine in terms of vulnerability [11-14].

## Case presentation

We present the case of a 20-year-old female patient with no significant past medical history or occupational exposures, who presented to the neurosurgery clinic with symptoms of progressive paraparesis and urinary retention syndrome. 

The patient's symptoms began insidiously approximately one month prior to presentation, with a progressive decline in motor function leading to a 3/5 score on the MRC scale for muscle strength and development of urinary retention syndrome. Clinical examination revealed a sensory level at T10, bilateral extensor plantar responses, bilateral hyperreflexia of the lower limbs, and catheterized urinary retention. No pathological changes were observed biologically.

**Figure 1:**
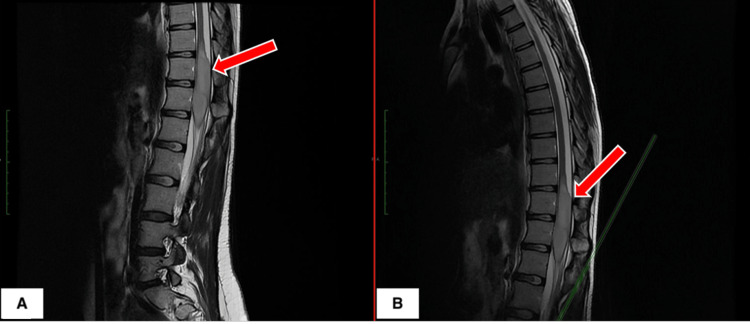
Preoperative MRI T2 sequence, (A) thoracic and (B) lumbar spinal cord, sagittal section showing T10-L1 intramedullary lesion with infiltrative character

**Figure 2:**
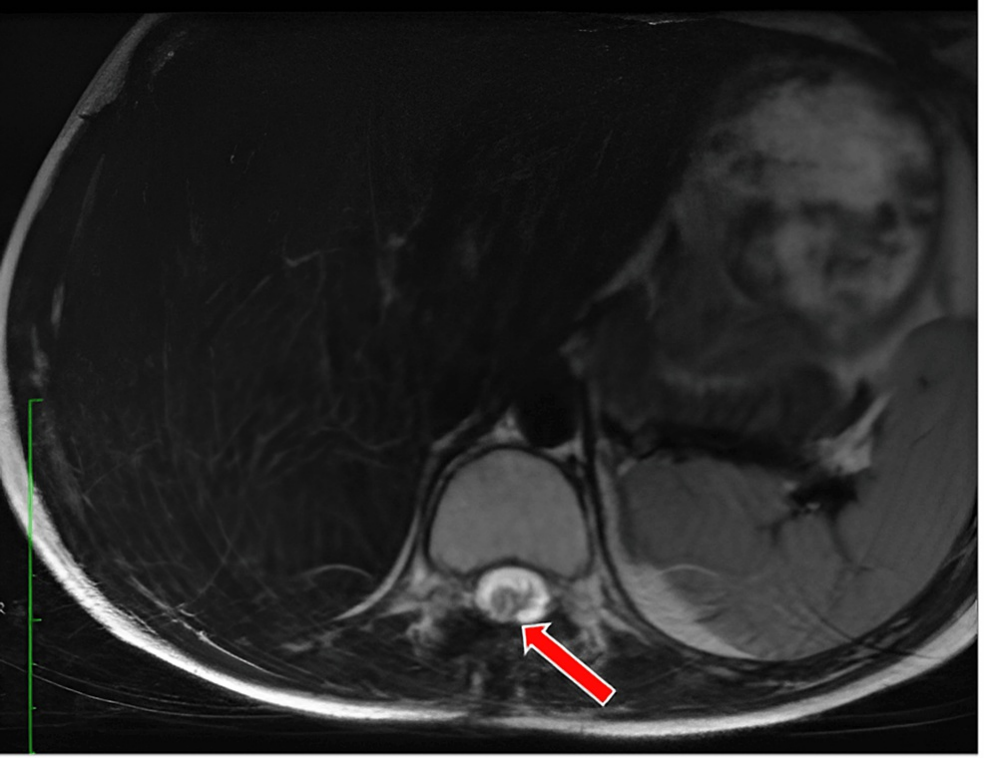
Preoperative MRI T2 sequence, lumbar spinal cord, axial section showing intramedullary lesion with infiltrative character

**Figure 3:**
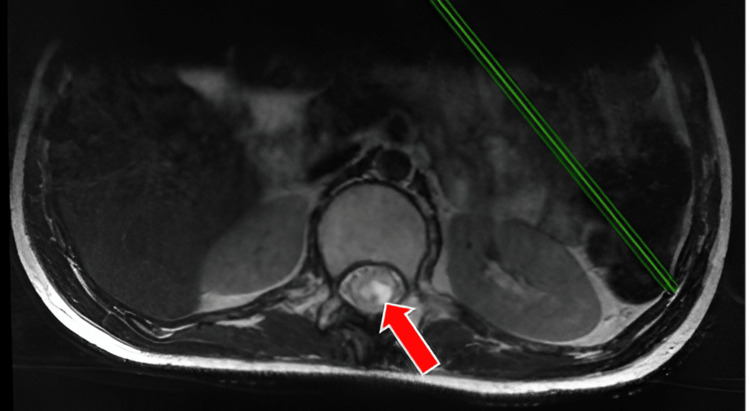
Preoperative MRI T2 sequence, lumbar spinal cord, axial section showing intramedullary lesion with infiltrative character

Magnetic Resonance Imaging (MRI) of the dorsolumbar spine, performed natively, demonstrated an intramedullary lesion spanning T10-L1 with an infiltrative character suggestive of ependymomas, fibrillary astrocytoma, glioblastoma, or syringomyelia (Figures 1A, 1B). This imaging finding was essential in guiding the subsequent surgical approach and management strategy (Figures 2, 3).

**Figure 4:**
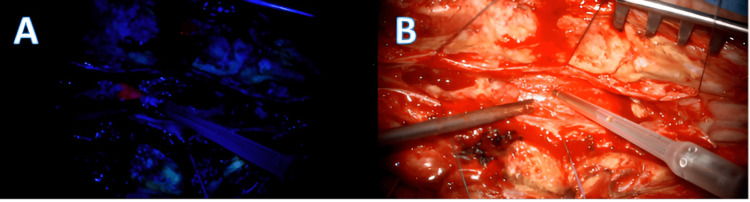
Intraoperative view of surgery. (A) Fluorescence-guided surgery. (B) Microscopic-guided surgery.

**Figure 5:**
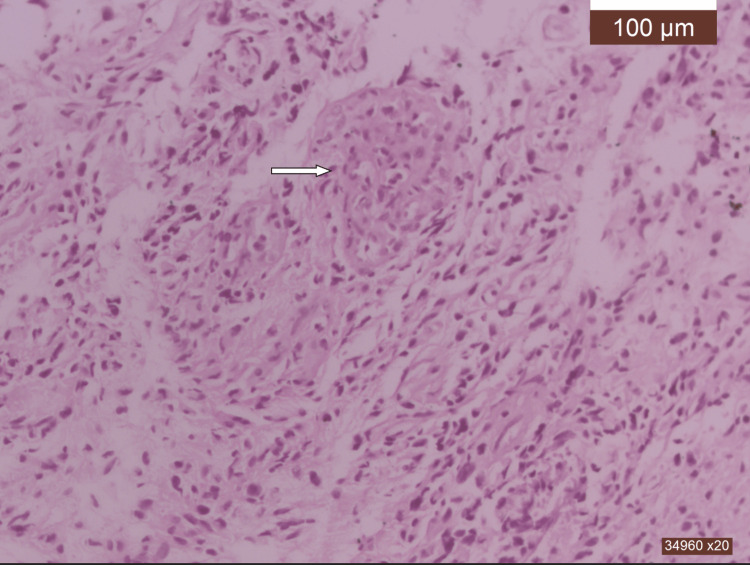
Medullary glioblastoma (HE, x40) Medullary glioblastoma, IDH1 wild-type, grade 4. Densely cellularized tumor proliferation, composed of diffusely arranged anaplastic astrocytes, on a fibrillar background. A vessel with glomeruloid-type endothelio-pericytic proliferation can be identified intratumorally (arrow).

**Figure 6:**
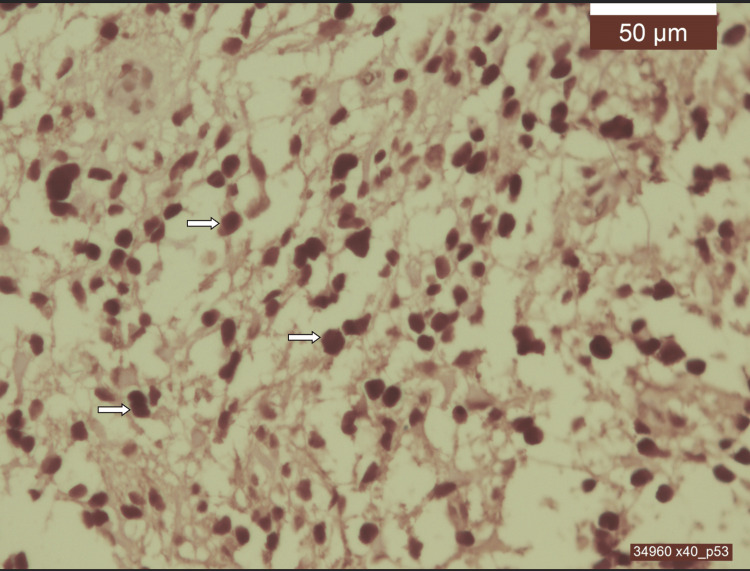
IHC identifies the presence of p53 protein, showcasing TP53 mutation Medullary glioblastoma, IDH1 wild-type, grade 4. p53 positive in 100% of tumor cell nuclei - brown staining of positive nuclei (arrows) (IHC, x40).

**Figure 7:**
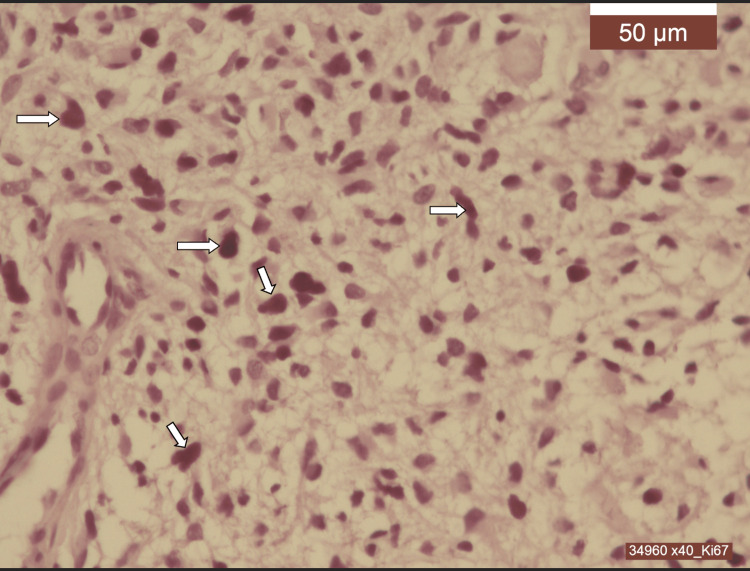
Binding index of KI-67 Medullary glioblastoma, IDH1 wild-type, grade 4. Ki67 binding index has very high values ​​(25%) - brown nuclear staining (arrows).

The patient underwent a T10-L1 laminectomy, median durotomy, and dissection of the posterior median sulcus. This revealed a solid intramedullary lesion without a clear plane of separation but with infiltrative areas. Resection was achieved using Cavitron Ultrasonic Surgical Aspirator (CUSA) under electrophysiological monitoring (Figures 4A, 4B). Postoperative recovery was notable for improvement in motor deficit and resolution of urinary retention syndrome. The final histopathological diagnosis confirmed IDH1 wild-type glioblastoma (Figure 5), with TP53 mutation (Figure 6) and high Ki-67 mitotic index (25%) (Figure 7). The patient received adjuvant chemotherapy with a daily dose of 120 mg temozolomide (TMZ) for six weeks and concomitant radiotherapy (RT) with 2 Gy fractions daily, five days a week, the total being 60 Gy over six weeks. The tumor demonstrated an initial positive response to treatment.

After a loss to follow-up, the patient returned after two years with paraplegia and worsening urinary retention, now scoring 0/5 on the MRC scale. Contrast-enhanced MRI revealed tumor recurrence with dorsal and lumbar extension. Given the rapid progression of motor deficits and the grave prognosis, a decision was made for cordectomy up to T6, augmented by gliolan for enhanced visualization of the tumor infiltrate, aiming for supramarginal resection. No tumor cells were detected in the CSF collected intraoperatively.

A T6-L3 laminectomy and resection of the infiltrated dorsal spinal cord and intracanal tumor mass were performed, followed by a watertight dura suture at T6. Postoperative imaging confirmed complete lesion resection. The patient underwent chemotherapy with TMZ and an MRI covering the entire neuraxis. Six months postoperatively, a transpedicular fixation from T5 to L3 was performed using O-arm. Seven months postoperatively, MRI revealed two new lesions without symptomatic manifestation.

The patient developed slowly progressive cognitive disturbances and somnolence, opting with her family for conservative and supportive treatment. She suffered a cardiorespiratory arrest unresponsive to resuscitation approximately 10 months post-cordectomy. This case underscores the aggressive nature of glioblastoma and the complexities involved in managing spinal cord involvement, highlighting the importance of a multidisciplinary approach to care and the need for ongoing research into more effective treatments for this devastating condition.

## Discussion

Glioblastoma has a generally very low average survival rate, of approximately 15 months post diagnosis [8,15]. In the case of this tumor residing in the spinal cord, the overall survival (OS) is between six and 16 months post diagnosis [12,16,17]. An analysis performed by Shen et al. proved that thoracolumbar glioblastoma has an approximate OS of 21.1 months [18]. Thus, our case showcases the fact that with modern treatment techniques, survival can be increased significantly, with our patient surviving for 34 months after being diagnosed.

Primary spinal cord glioblastomas may emerge de novo as primary grade 4 astrocytomas or evolve from astrocytomas of a lower grade. Moreover, they exhibit histological characteristics akin to those of supratentorial glioblastomas, encompassing nuclear atypia, mitotic activity, palisading necrosis, microvascular or glomeruloid vascular proliferation, and increased levels of Ki-67 antigen [9].

Prior research has indicated that TMZ did not significantly enhance survival outcomes in primary spinal cord glioblastoma. Nonetheless, evidence suggests that a combinatory approach involving postoperative RT alongside TMZ chemotherapy might facilitate improved survival rates for spinal cord glioblastoma patients [19,20]. Studies conducted by Chamberlain et al. and Kaley et al. have demonstrated that Bevacizumab, when used as a salvage therapy, can elicit responses that correlate with clinical amelioration in patients suffering from recurrent spinal cord glioblastoma and high-grade spinal cord gliomas [21,22].

In the treatment of glioblastoma, there is a peculiar interest in the development of nanomaterials, to be more exact biodegradable membranes that can be surgically implanted in order to try and treat this neoplasm. Tseng et al. conducted a research study that entailed the fabrication of a biodegradable nanofibrous membrane composed of poly[(d,l)-lactide-co-glycolide] through the electrospinning technique. This membrane was engineered to facilitate a prolonged release of carmustine (BCNU). The results suggest that the biodegradable, nanofibrous structures were capable of maintaining elevated levels of BCNU release within the cerebral cavity of rat models for a duration exceeding six weeks. Additionally ensured comprehensive coverage of the affected areas post-tumor resection. This attribute enhances the efficiency of drug delivery while preserving the normal functional integrity of the brain. Histopathological evaluations further indicated an absence of significant inflammatory responses in the brain tissues treated with these membranes, suggesting their biocompatibility and potential therapeutic efficacy in the context of post-surgical cancer treatment [23]. The development of nanomaterials, we believe, is a promising avenue of research that should be further explored.

## Conclusions

This case of a 20-year-old woman with progressive paraparesis diagnosed with spinal glioblastoma highlights the challenges of managing primary spinal cord cancers. Despite glioblastoma mainly being a brain tumor known for its aggressive behavior and poor prognosis, its presence in the spinal cord poses even greater therapeutic hurdles. Documenting each spinal glioblastoma case is crucial for understanding and improving treatment approaches. The patient's initial positive response to aggressive treatment, extending her survival to 34 months, underscores the importance of multidisciplinary care. However, the tumor's recurrence and her eventual shift to supportive care reflect the severe outcomes often associated with this diagnosis. This case adds valuable insight into spinal glioblastoma, emphasizing the need for ongoing research and advances in neuro-oncology.
